# Targeting Inflammation: Cytosporone B Modulates Imatinib-Driven Biochemical Alterations in Rat Heart

**DOI:** 10.3390/ijms262010018

**Published:** 2025-10-15

**Authors:** Denise Börzsei, András Nagy, Viktória Kiss, Zoltán Virág, Gyöngyi Kis, Nikoletta Almási, Szilvia Török, Médea Veszelka, Csaba Varga, Renáta Szabó

**Affiliations:** Department of Physiology, Anatomy, and Neuroscience, Faculty of Science and Informatics, University of Szeged, H-6726 Szeged, Hungary; nagy.andras.levente99@gmail.com (A.N.); k.viktoria007@gmail.com (V.K.); almasi@expbio.bio.u-szeged.hu (N.A.); veszmed@bio.u-szeged.hu (M.V.);

**Keywords:** Imatinib, Cytosporone B, inflammation, heart

## Abstract

In recent decades, chemotherapy has significantly improved cancer survival, yet its adverse effects on non-cancerous tissues raise increasing concerns. In this context, growing attention has been focused on natural compounds that may be useful in mitigating the undesirable effects of chemotherapeutic agents. Here, we aimed to demonstrate that Cytosporone B (CsnB) is a potent agent for counteracting the cardiovascular effects induced by Imatinib. To this end, 12-week-old male Wistar rats were studied; they were divided into three groups as follows: (1) control, (2) Imatinib-treated (Imatinib: 60 mg/kg/day, per os), (3) Imatinib + CsnB-treated (CsnB: 5 mg/kg/day, i.p.). After the two-week-long experimental period, rats were euthanized. Their hearts were used for the following biochemical measurements: NADPH oxidase (NOX4), high mobility group box 1 (HMGB1), peptidylarginine deiminase 4 (PAD4), inducible nitric oxide synthase (iNOS) expression, tumor necrosis factor-alpha (TNF-α) level, and myeloperoxidase (MPO) activity. Imatinib caused a marked upregulation of key inflammatory and oxidative markers, including HMGB1, TNF-α, MPO, iNOS, PAD4, and NOX4 in cardiac tissue; however, CsnB treatment mitigated these elevations, implying its role in opposing Imatinib-induced inflammatory and oxidative processes in the heart. Our findings suggest that CsnB holds promise as a cardioprotective agent capable of modulating Imatinib-induced adverse cardiac effects.

## 1. Introduction

Chemotherapeutic agents remain one of the most effective tools in modern oncology, saving countless lives by preventing the progression of various cancers. However, despite its life-prolonging benefits, serious side effects can be a cause for concern. Evidence substantiates an indisputable link between certain chemotherapeutic drugs and long-term cardiovascular complications, posing new challenges in cancer therapy [1,2]. While anthracyclines and platinum-based agents are classical examples of cardiotoxic drugs, more recently targeted agents, including tyrosine kinase inhibitors (TKIs), have also been implicated in adverse cardiovascular outcomes [3]. Imatinib (Imtb)—known as the first FDA-approved TKI—is generally considered to have a favorable safety profile; however, beyond its established role in the cell cycle, expanding evidence indicates it also exerts off-target effects on cardiac tissue. Studies have documented that Imtb potentially has cardiotoxic effects, which are mediated through complex interactions involving mitochondrial dysfunction, oxidative stress [4], and inflammation [5] in cardiomyocytes. One of the key cellular processes potentially targeted by Imtb is the mitochondrial respiratory chain in myocardial cells, whose impairment leads to the upregulation of NADPH oxidase 4 (NOX4) activity and an enhanced inflammatory response [4]. This leads to the activation of pro-inflammatory cytokines, including tumor necrosis factor-alpha (TNF-α) and high mobility group box 1 (HMGB1). These inflammatory mediators promote the production of peptidylarginine deiminase 4 (PAD4), one of the key players in neutrophil extracellular trap (NET) formation. Myeloperoxidase (MPO), which is fundamental to the assembly of NET, further aggravates oxidative damage and amplifies tissue injury [6]. Consequently, oxidative and inflammatory injuries in the heart induce inducible nitric oxide synthase (iNOS) expression that further exacerbates adverse cardiac processes [7].

In light of the involvement of multiple inflammatory and oxidative mediators in Imtb-induced cardiac injury, these pathways may serve as critical intervention points for therapeutic modulation aimed at preserving cardiac function during cancer treatment. As survival rates for cancer patients are increasing, there is a growing need to find alternative therapeutic modalities to reduce the toxic side effects of chemotherapy drugs. In addition to pharmacological drugs, more and more attention is being focused on natural substances with cardioprotective effects through cell protection and anti-inflammatory mechanisms. Cytosporone B (CsnB), with great potential, is an octaketide which was isolated from an endophytic fungus *Dothiorella* sp. HTF3 [8]. CsnB is a natural agonist for nuclear orphan receptor Nur77, through which it modulates countless biological processes, including inflammation. Beyond its role in inflammation, Nur77 has been implicated in multiple cardiovascular and metabolic processes, including the mediation of cardiomyocyte apoptosis [9], glucose utilization in muscle [10], and vascular remodeling [11]. By acting as a natural agonist of Nur77, CsnB may therefore exert a broader spectrum of protective effects on cardiac tissue, extending beyond the regulation of inflammatory pathways. Although its anti-inflammatory properties have been demonstrated in various disease models, its potential role in Imtb-induced cardiac injury has not yet been elucidated [12,13,14].

Therefore, this study aims to investigate the potential cardioprotective effects of CsnB in the context of Imtb-induced cardiac alterations. Accordingly, we intended to determine whether CsnB can diminish the upregulation of key pathological markers after Imtb treatment—including TNF-α, MPO, NOX4, HMGB1, PAD4, and iNOS—and recover cardiac homeostasis. To our best knowledge, this study is the first that seeks to investigate this tightly interwoven network of oxidative stress and inflammation in the mechanism of action of CsnB.

## 2. Results

### 2.1. Cardiac NOX4 Expression

**Figure 1:**
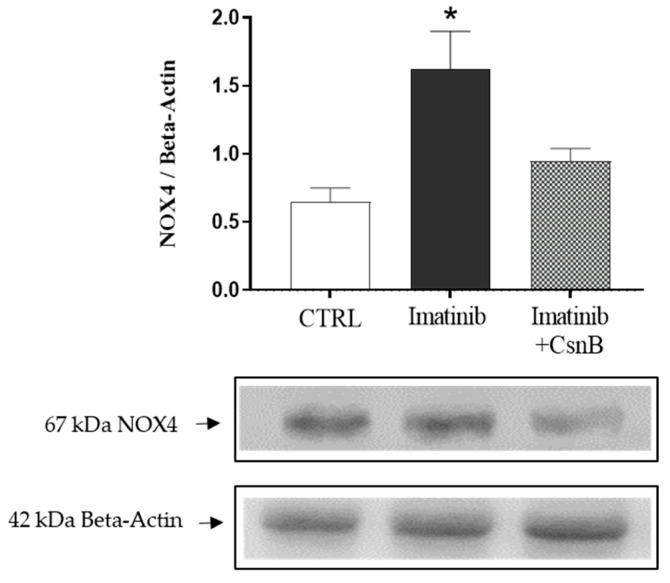
The effects of CsnB treatment on cardiac NOX4 concentration. Results shown as means ± S.E.M. n = 4–6, Kruskal–Wallis one-way analysis and Dunn’s method, * *p* < 0.05 statistical comparison between CTRL and Imatinib groups, CTRL = control, CsnB = Cytosporone B, NOX4 = NADPH oxidase.

To explore the initiation of oxidative stress in Imtb-induced cardiac injury, we quantified NOX4 expression, a major enzymatic source of reactive oxygen species in cardiomyocytes. Imtb treatment resulted in a clear increase in NOX4 expression compared to the control group, indicative of enhanced oxidative burden. CsnB administration, on the other hand, efficiently suppressed NOX4 levels, suggesting its antioxidant potential in the myocardium. Data are presented in Figure 1.

### 2.2. TNF-α Concentration and HMGB1 Expression of the Heart

**Figure 2:**
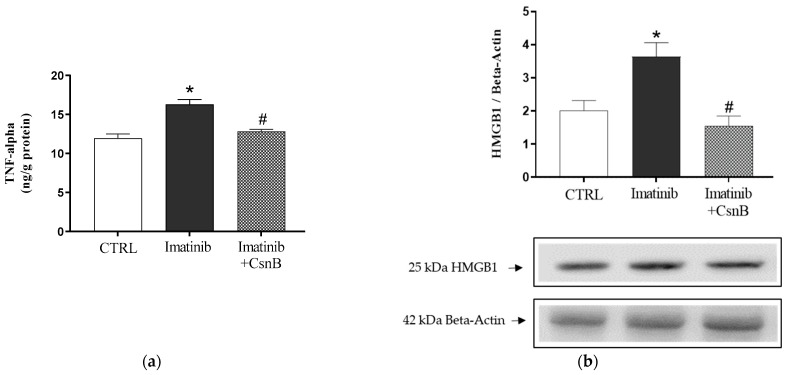
(**a**) The effects of CsnB treatment on cardiac TNF-α concentration (TNF-α; expressed as ng/g protein). Result shown as means ± S.E.M. n = 7–8, one-way ANOVA and Tukey post hoc test, * *p* < 0.05 statistical comparison between CTRL and Imatinib groups, #: *p* < 0.05 statistical comparison between Imatinib and Imatinib + CsnB groups. (**b**) The effects of CsnB treatment on cardiac HMGB1 expression. Results shown as means ± S.E.M. n = 5–6, one-way ANOVA and Tukey post hoc test, * *p* < 0.05 statistical comparison between CTRL and Imatinib groups, #: *p* < 0.05 statistical comparison between Imatinib and Imatinib + CsnB groups, CTRL = control, CsnB = Cytosporone B, HMGB1 = high mobility group box 1, TNF-α = tumor necrosis factor-alpha.

To assess the inflammatory status of the heart, TNF-α concentration was determined. As Figure 2a shows, TNF-α expression was significantly elevated in the Imatinib group, suggesting a pro-inflammatory cardiac environment. CsnB co-treatment significantly decreased TNF-α levels, bringing them closer to control values. As TNF-α is a central mediator of inflammation, its reduction suggests that CsnB has anti-inflammatory effects.

As shown in Figure 2b, Western blot analysis revealed that HMGB1 expression was significantly elevated in the Imtb group relative to control animals, reflecting increased cellular stress and inflammation as a result of the aforementioned chemotherapeutic agent. Co-administration of CsnB together with Imtb significantly attenuated this upregulation.

### 2.3. MPO Activity and PAD4 Expression of the Heart

**Figure 3:**
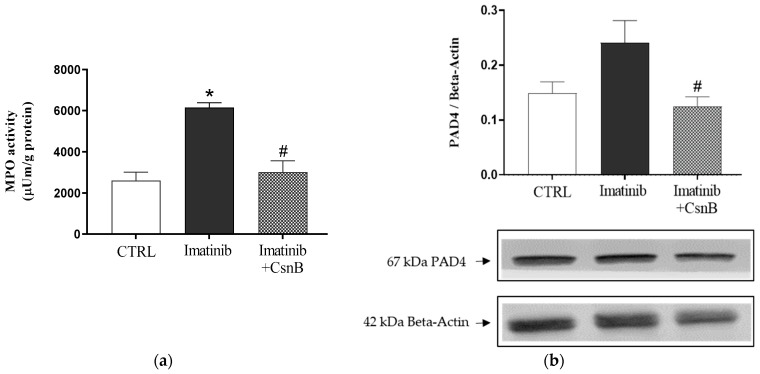
(**a**) The effects of CsnB treatment on cardiac MPO activity (MPO; expressed as µU/mg protein). Results shown as means ± S.E.M. n = 6–8, one-way ANOVA and Tukey post hoc test, * *p* < 0.05 statistical comparison between CTRL and Imatinib groups, #: *p* < 0.05 statistical comparison between Imatinib and Imatinib + CsnB groups. (**b**) The effects of CsnB treatment on cardiac PAD4 expression. Results shown as means ± S.E.M. n = 5–6, one-way ANOVA and Tukey post hoc test, #: *p* < 0.05 statistical comparison between Imatinib and Imatinib + CsnB groups, CTRL = control, CsnB = Cytosporone B, MPO = myeloperoxidase, PAD4 = peptidylarginine deiminase 4.

Similar to TNF-α concentration, MPO activity was significantly increased in the Imtb-treated group compared to control animals, indicating enhanced neutrophilic infiltration and inflammation in the myocardium. On the other hand, CsnB treatment resulted in a significant decrease in MPO activity compared to Imtb-treated groups, suggesting a key role in alleviating the inflammatory burden. Data are presented in Figure 3a.

To assess the role of NET formation and chromatin modification in cardiac inflammation, we evaluated PAD4 expression in heart tissue. While PAD4 expression was elevated in the Imtb group, consistent with enhanced inflammatory mechanisms, CsnB treatment significantly lowered PAD4 levels, indicating a potential mechanism by which CsnB may attenuate neutrophil-driven cardiac damage. The changes in PAD4 expression are shown in Figure 3b.

### 2.4. Cardiac iNOS Expression

**Figure 4:**
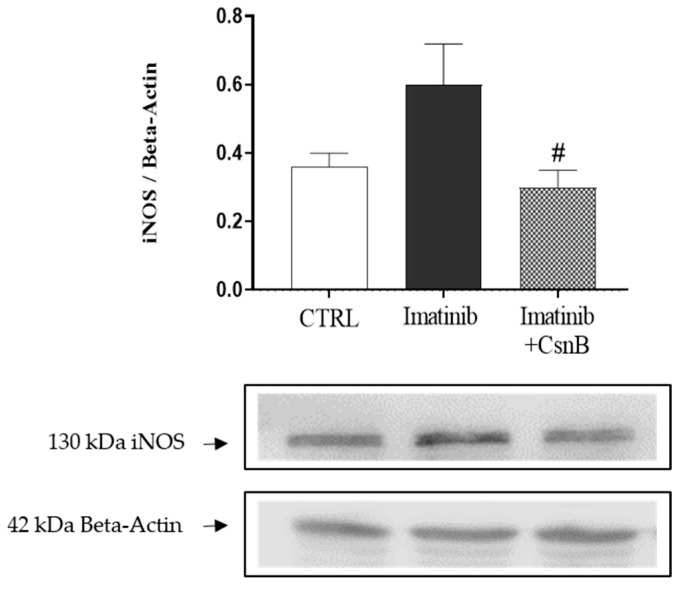
The effects of CsnB treatment on cardiac iNOS expression. Results shown as means ± S.E.M. n = 4–6, Kruskal-–Wallis one-way Analysis and Dunn’s method, #: *p* < 0.05 statistical comparison between Imatinib and Imatinib + CsnB groups, CTRL = control, CsnB = Cytosporone B, iNOS = inducible nitric oxide synthase.

To gain insight into the nitrosative stress response and pro-inflammatory signaling in the myocardium, we assessed the expression of iNOS. Cardiac iNOS expression was remarkably upregulated in the Imtb-treated group compared to the control group. Nevertheless, CsnB administration led to a significant reduction in iNOS levels, in comparison to the Imtb group. Suppression of iNOS suggests that CsnB may contribute to redox homeostasis and inflammation control under Imatinib-induced stress conditions. Data are presented in Figure 4.

## 3. Discussion

Chemotherapy-associated cardiac damage is a growing concern in oncology, particularly in long-term cancer survivors. Imtb—a commonly used tyrosine kinase inhibitor— stands as a cornerstone modality in cancer treatment and has been considered relatively safe from multiple standpoints [15]. However, increasing clinical and experimental data suggest that Imtb may exert off-target effects affecting cardiac tissue [16,17,18]. First of all, Imtb has been shown to induce mitochondrial dysfunction via structural changes, such as effaced mitochondrial cristae or mitochondrial swelling, as well as through endoplasmic reticulum stress and collapse of the mitochondrial membrane potential, leading to increased oxidative stress and apoptotic cell death [19,20,21]. In addition to oxidative stress, myocardial apoptosis is also associated with Imtb-induced increase in proinflammatory signaling mediated by TNF-α, interleukin-6, and mitogen-activated protein kinase pathways [5]. Thus, it is safe to say that Imtb treatment might present a risk to the integrity of cardiac tissue and to healthy cardiovascular function. Our study supports this hypothesis by demonstrating that Imtb treatment leads to the upregulation of several pro-inflammatory and oxidative stress markers in cardiac tissue, including NOX4, TNF-α, HMGB1, PAD4, MPO, and iNOS. Importantly, co-administration of CsnB effectively attenuated the elevation of these markers, suggesting its potential cardioprotective properties.

In the current study, rats received Imtb at a dose of 60 mg/kg for two weeks, every day. By selecting this dose based on previous studies, our goal was to model a potentially deleterious, inflammatory condition in the heart. Recent studies have shown that Imtb can promote mitochondrial dysfunction, oxidative stress, and inflammation in cardiomyocytes, resulting in cardiac impairment in certain cases. Herman et al. in particular found cardiac lesions and cardiomyocyte apoptosis after two weeks of low-dose Imtb treatment in rats [22]. Similarly, a study by Kerkela et al. demonstrated the cardiotoxic effects of low-dose Imtb, mediated by mitochondrial and endoplasmic reticulum stress [19]. Building on these observations, in the present study we aimed to gain a deeper understanding of the inflammatory processes triggered by Imtb in cardiac tissue. Cardiac inflammation is the earliest event in the progression toward structural and functional heart damage, and shedding light on its molecular drivers is crucial for identifying preventive and therapeutic strategies. Inflammation induced by chemotherapeutic agents arises from a highly complex and multifactorial interplay of molecular and cellular mechanisms. One of the earliest molecular events in this process is the upregulation of NOX4, a major source of ROS in cardiomyocytes, which sets in motion a cascade of redox-sensitive pathways leading to the activation of multiple pro-inflammatory mediators. NOX4 is abundantly expressed in the heart, primarily located in the mitochondria [23]. It is a unique member of the NOX family, since it is constitutively active. Functionally, NOX4 primarily generates hydrogen peroxide (H_2_O_2_) rather than superoxide, which enables it to diffuse across membranes and modulate signaling in distant cellular compartments. In cardiomyocytes, excessive NOX4 activity has been associated with mitochondrial dysfunction, endoplasmic reticulum stress, and apoptotic signaling, all of which contribute to structural and functional cardiac injury [24,25]. Although results from previous studies linking Imtb treatment to NOX4 activation in the heart are currently lacking, it is well-described that Imtb can induce mitochondrial ROS production, oxidative stress, and inflammatory responses in cardiac tissue [19,22]. Given the well-established role of NOX4 as a central upstream regulator of inflammatory cascades, it is plausible that NOX4 may contribute to the oxidative and inflammatory milieu observed during Imtb-induced cardiac injury. Our results clearly show that as a result of the two-week-long Imtb treatment, NOX4 levels were significantly higher compared to the control group. Increased NOX4 expression promotes the production of pro-inflammatory cytokines such as TNF-α and other inflammatory mediators like HMGB1. In addition, TNF-α in fact could initiate the upregulation of NOX4 itself [26], creating a feed-forward loop of oxidative stress and inflammation within the cardiac tissue. Elevated TNF-α amplifies the inflammatory response by promoting leukocyte adhesion, increasing endothelial permeability, and inducing apoptosis in cardiomyocytes. HMGB1, released as a result of stress stimuli under oxidative and inflammatory conditions, functions as a damage-associated molecular pattern (DAMP) that binds to receptors such as TLR4 and RAGE, further sustaining inflammation and immune cell recruitment to the myocardium. Our findings have also underpinned that as a result of Imtb treatment, a severe inflammatory state was manifested, as reflected by the markedly elevated levels of TNF-α and HMGB1.

Cardiac inflammatory reactions characterized by accumulated proinflammatory markers further contribute to cardiac injury. Elevated TNF-α and HMGB1 promote leukocyte recruitment and neutrophil activation, which in turn facilitate the induction of PAD4. PAD4 plays a central role in the citrullination of histones, a key step in NET formation. Although NETs are primarily essential for protection against various pathogens, the latest findings indicate that they also play an important role in the pathophysiology of various non-infectious diseases [27]. Du et al. proved that the inhibition of PAD4 can moderately preserve myocardium integrity after myocardial infarct (MI) and successfully protects cardiomyocytes from MI-induced NET formation and cytokine secretion [28]. The structure of NETs contains further neutrophil-derived proteins such as MPO. This abundant granule enzyme is stored in the azurophilic granules of the neutrophils and during its operation it generates hypochlorous acid and other reactive oxidants that contribute to tissue damage [29]. MPO translocates to the nucleus, where, in cooperation with neutrophil elastase, it contributes to chromatin decondensation and the subsequent release of neutrophil extracellular traps. The combined activation of PAD4 and MPO thus establishes a self-sustaining cycle of NET formation, oxidative damage, and cytokine release, driving further cardiac inflammation and injury [30]. Recent studies indicate that MPO serves as a critical mediator connecting oxidative stress with inflammatory pathways, thereby contributing to the development of cardiac pathologies [31,32,33]. Given the role of PAD4 and MPO in amplifying the inflammatory response within cardiac tissue, our goal was to assess whether treatment with Imtb, as a commonly used chemotherapeutic agent, modulates these inflammatory and oxidative parameters, thereby contributing to the pathogenesis of cardiac alterations. Our current findings show that as a result of Imtb administration PAD4 levels were elevated in the heart compared to the CTRL group. Furthermore, MPO activity observed in Imtb-treated groups were also significantly higher in comparison to CTRL animals.

Additionally, our findings tend to highlight the contribution of iNOS to Imtb-induced cardiac inflammation. iNOS is minimally expressed under physiological conditions, but during oxidative stress and inflammatory processes, it becomes strongly upregulated in cardiomyocytes, endothelial cells, and infiltrating immune cells [34]. Once it is activated, it produces an excessive amount of nitric oxide (NO), which can be cytotoxic. iNOS produces superoxide anions as well (along with superoxide anions derived from NOX4 activity or MPO-generated oxidants) which further exacerbates oxidative stress and initiates nitrosative damage to proteins, lipids, and mitochondrial DNA, thereby further impairing cardiac function [35]. Mechanistically, TNF-α and HMGB1, both elevated in our Imtb-treated groups, are well-documented transcriptional inducers of iNOS through NF-κB-dependent pathways. In parallel, MPO-derived oxidants and PAD4-dependent NET formation create a microenvironment that sustains inflammatory signaling and facilitates iNOS induction. In line with this mechanistic framework, our results show that iNOS expression was increased in cardiac tissue after two weeks of Imtb administration, further supporting the establishment of a highly pro-oxidant, pro-inflammatory milieu.

**Figure 5:**
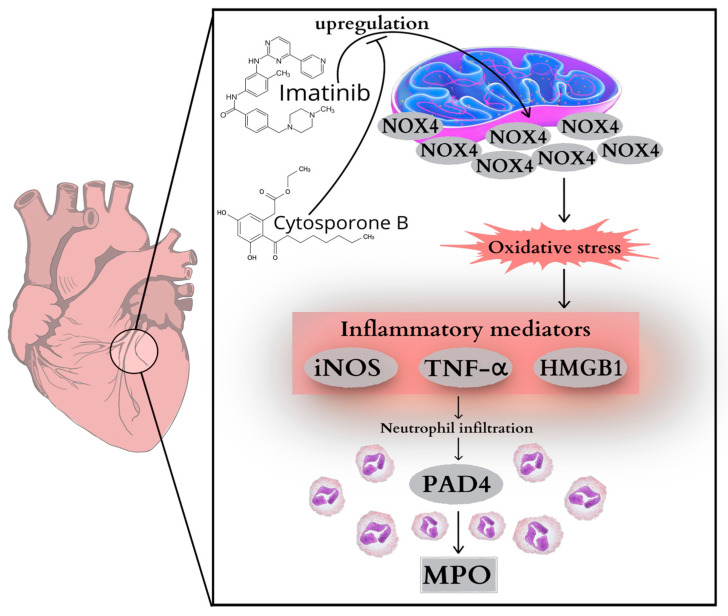
Effects of Cytosporone B on Imatinib-induced cardiac inflammation. HMGB1 = high mobility group box 1, iNOS = inducible nitric oxide synthase, MPO = myeloperoxidase, NOX4 = NADPH oxidase, PAD4 = peptidylarginine deiminase 4, TNF-α = tumor necrosis factor-alpha.

CsnB is one of the first described natural agonists for orphan receptor Nur77. Nur77 belongs to the nuclear receptor subfamily 4 group A (NR4A), which has been shown to act as a key regulator of multiple processes, including inflammation, metabolism, or stress response [12,13]. Exploiting the characteristics of Nur77 may pave the way for new therapeutic strategies against various inflammatory diseases. A series of studies has shown that Nur77 has anti-inflammatory properties through direct interactions with NF-κB in different organs [36,37,38]; moreover, a Nur77 knockout model studied by Kurakula et al. proved increased inflammatory responses as well [39]. The rationale for selecting CsnB as a therapeutic candidate in our study is based on its well-documented anti-inflammatory and cytoprotective properties linked to Nur77. CsnB, a natural agonist of Nur77, has been investigated in various pathological contexts, including cardiovascular, hepatic, and neurodegenerative disorders, where it demonstrated the ability to attenuate inflammatory responses and mitigate cellular damage. Growing evidence supports the fact that CsnB can suppress pro-inflammatory cytokine production, inhibit oxidative stress pathways, and enhance cellular resilience under pathological conditions [12,13,14]. These findings collectively highlight CsnB as a promising candidate for mitigating Imtb-induced cardiac injury. Consistent with these observations, our findings demonstrate that CsnB treatment effectively diminished Imtb-induced elevations in key inflammatory and oxidative stress markers, including NOX4, TNF-α, HMGB1, PAD4 concentrations, MPO activity, and iNOS levels. These findings strengthen the notion that CsnB exerts cardioprotective effects by targeting multiple interconnected points of the inflammatory–oxidative network activated by Imtb (Figure 5). Presumably, CsnB may serve as a promising therapeutic strategy to counteract the cardiac-associated inflammatory and cytotoxic processes following Imtb treatment.

## 4. Materials and Methods

### 4.1. Animals

**Figure 6:**
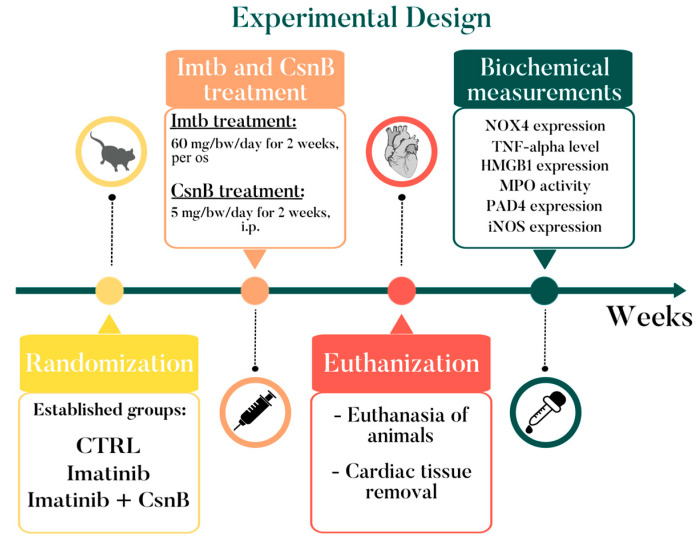
Experimental design of the study. CTRL = control, CsnB = Cytosporone B, HMGB1 = high mobility group box 1, iNOS = inducible nitric oxide synthase, i.p. = intraperitoneal, MPO = myeloperoxidase, NOX4 = NADPH oxidase, PAD4 = peptidylarginine deiminase 4, TNF-alpha = tumor necrosis factor-alpha.

All animal procedures were approved by the National Scientific Ethical Committee on Animal Experimentation (permission No. XX./3205/2022; date of approval: 19 December 2022) and correspond to the ARRIVE guidelines. Animals were purchased from Animalab Ltd., Vác, Hungary, and housed according to the Directive 2010/63/EU regulations, namely at 22 °C, with a 12 h light/dark cycle and free access to chow and tap water. At the beginning of the study, twelve-week-old male Wistar rats were randomly assigned into three groups as follows: control, non-treated (CTRL), Imatinib-treated (Imtb), and Imtb + Cytosporone B-treated (Imtb + CsnB). To induce cardiac inflammation, animals were given 60 mg/kg Imtb (Imtb, Sandoz, Basel, Switzerland) per os, every day for two weeks. The dose of Imtb was chosen to be within the clinically recommended range of 400–800 mg/day for a 70 kg adult, as outlined in therapeutic guidelines [40,41]. To clarify the potentially cell-protective effect of CsnB, animals in the third group were given 5 mg/kg CsnB [42] (HY-N2148, MedChemExpress, Monmouth Junction, NJ, USA) i.p. daily for two weeks in addition to the Imtb treatment. At the end of the experimental period, rats from all three groups were euthanized using thiopental i.p. (100 mg/kg, B. Braun Medical SA, Barcelona, Spain). Subsequently, their hearts were removed for further biochemical testing (Figure 6).

### 4.2. Determining Cardiac HMGB1, PAD4, NOX4, and iNOS Expressions

After the experimental period, heart tissue samples were homogenized (3 × 10 s) in ice cold RIPA buffer (Merck Millipore, Burlington, MA, USA) with an ultrasonic homogenizer (UP-100H, Hielscher Ultrasonics, Teltow, Germany). This was followed by centrifugation at 15,000× *g* for 10 min at 4 °C and collection of supernatants. Afterwards, total protein concentration was measured with a Pierce Bicinchoninic Acid (BCA) protein assay kit (Pierce, Thermo Fisher Scientific). Based on this, equal amounts from each sample (50 µg) were loaded onto 10% sodium dodecyl sulfate-polyacrylamide gel (SDS-PAGE) for 2 h electrophoresis at 90 V. After separation, proteins were transferred to a nitrocellulose membrane via 2.5-h electroblotting at 35 V. The membranes were then dyed with Ponceau solution, washed with Tris-buffered saline containing 0.1% Tween 20 (TBS-T), and incubated overnight with 5% nonfat dry milk powder or 5% bovine serum albumin (BSA) dissolved in TBS-T in order to prohibit nonspecific binding. Following incubation, the blots were washed for 3 × 10 min, and incubated at room temperature for two hours with the following primary antibodies: anti-HMGB1 (1:1000, ab79823, Abcam, Cambridge, UK), anti-PAD4 (1:1000, 17373-1-AP, Proteintech, Manchester, UK), anti-NOX4 (1:1000, ab133303, Abcam, Cambridge, UK), and anti-iNOS (1:500, ab3523, Abcam, Cambridge, UK). After that, blots were incubated with polyclonal goat anti-rabbit (1:5000, Dako Agilent, Santa Clara, CA, USA) at room temperature for one hour. β-actin served as control to demonstrate that equal amounts of protein were loaded in each lane. Similar to our target proteins, membranes were incubated with primary β-actin antibody (1:10,000, ab20272, Abcam, Cambridge, UK) at room temperature for two hours, followed by incubation with polyclonal rabbit anti-mouse secondary antibodies conjugated with horseradish peroxidase (1:5000, Dako Agilent, Santa Clara, CA, USA) for one hour. Protein determination was carried out with MagicMark XP Western Protein Standard (Invitrogen, Thermo Fisher Scientific, Waltham, MA, USA), using a standard consisting of nine recombinant proteins with molecular weights ranging between 20 kDa and 220 kDa. The bands were visualized by Li-Cor Odyssey XF imaging system (LiCorbio, Lincoln, NE, USA), developed using an enhanced chemiluminescence system (ECL Plus, Amersham Pharmacia Biotech., Buckinghamshire, UK), and analyzed using Quantity One Software version 4.5 (Bio-Rad Laboratories, Hercules, CA, USA). Results were normalized to β-actin and presented as relative expressions.

### 4.3. Measurement of Cardiac TNF-α Concentration

Cardiac tissue samples were homogenized (Benchmark D1000 Homogenizer, Sayreville, NJ, USA, 2 × 10 s) in phosphate buffer (PBS, pH 7.4), centrifuged at 2500 rpm for 20 min at 4 °C, and supernatants were collected to analyze TNF-α concentrations. Measurements were carried out using an enzyme-linked immunosorbent assay (ELISA) kit purchased from GenAsia Biotech Co. (Shanghai, China). Briefly, each well on the microplate contained either 100 µL standard diluent (blank wells), 50 µL standard solution (with biotin-labelled antibodies already mixed in), and 50 µL Streptavidin-HRP (standard wells) or 40 µL sample supernatant, 10 µL biotin-labelled antibodies, and 50 µL Streptavidin-HRP (sample wells). The microplates were incubated for 60 min at 37 °C and washed five times; then, 50 µL of chromogen A and B solutions were added to each well, followed by 10 min of incubation at 37 °C. Finally, 50 µL stop solution was added to each well, and absorbance (OD) was measured at 450 nm (Benchmark Microplate reader, Bio-Rad, Hercules, CA, USA). TNF-α concentrations were expressed as pg/mg protein.

### 4.4. Measurement of Cardiac MPO Enzymatic Activity

Cardiac tissue samples were homogenized (Benchmark D1000 Homogenizer, 2 × 10 s) in 0.5% hexadecyltrimethylammonium bromide (HETAB) and PBS (pH 7.4). Following homogenization, the samples went through four cycles of thawing and freezing with liquid nitrogen and were centrifuged at 10,000× *g* for 15 min at 4 °C. Using a 96-well microplate, 12 µL standard solution or sample supernatant was added to 280 µL o-dianisidine dihydrochloride. Adding 20 µL of hydrogen peroxide induced the color-changing reaction. After shaking the microplate for 30 s, MPO activity was measured spectrophotometrically (Benchmark Microplate reader, Bio-Rad, Hercules, CA, USA) at 490 nm, and values were expressed as µU/mg protein.

### 4.5. Statistical Analysis

Statistical analysis was performed using SigmaPlot 12 (Systat Software Inc., San Jose, CA, USA). Normality was checked with the Shapiro–Wilk test and for parametric distribution one-way ANOVA was used, followed by the Tukey post hoc test, while the Kruskal–Wallis test followed by the Tukey test was performed for nonparametric data. The obtained results are presented as mean values ± SEM. Significant differences were considered for all measurements where *p* values were found to be lower than 0.05.

## 5. Limitations

A key limitation of the present study is the absence of direct assessment of cardiac function, since our primary focus was on the biochemical analysis of inflammatory markers and their potential modulation by CsnB in the context of Imtb-induced cardiac inflammation. We recognize the need for further studies incorporating functional evaluations to support whether the observed biochemical alterations translate into clinically relevant improvements in cardiac health.

## Data Availability

All data used to support the findings of this study are included within the article.

## Abbreviations

The following abbreviations are used in this manuscript:

CsnB	Cytosporone B
CTRL	control
DAMP	damage-associated molecular pattern
ELISA	enzyme-linked immunosorbent assay
Imtb	Imatinib
iNOS	inducible nitric oxide synthase
MPO	myeloperoxidase
NADPH	nicotinamide adenine dinucleotide phosphate
NET	neutrophil extracellular trap
NO	nitric oxide
NOX4	NADPH oxidase
NR4A	nuclear receptor subfamily 4 group A
H_2_O_2_	hydrogen peroxide
HMGB1	high mobility group box 1
PAD4	peptidylarginine deiminase 4
PBS	phosphate buffer
TNF-α	tumor necrosis factor-alpha
